# CNN3 in glioma: The prognostic factor and a potential immunotherapeutic target

**DOI:** 10.1097/MD.0000000000027931

**Published:** 2021-11-19

**Authors:** Hao Xu, Song-shan Chai, Peng Lv, Jia-jing Wang

**Affiliations:** a Department of Neurosurgery, General Hospital of the Yangtze River Shipping, Wuhan, China; b Department of Neurosurgery, Union Hospital, Tongji Medical College, Huazhong University of Science and Technology, Wuhan, China; c Department of Neurosurgery, Suizhou Hospital, Hubei University of Medicine, Suizhou, China.

**Keywords:** biomarker, CNN3, gliomas, immunotherapeutic targets, prognostic factors

## Abstract

**Background:**

Gliomas are the most intrinsic type of primary intracranial tumors. The protein encoded by The calponin 3 (CNN3) has been proven to be a member of the calponin family. Its relationships with cervical cancer, colorectal cancer, gastric cancer, and colon cancer have been emphasized by several studies. Our research aims to explore the prognosis value and immunotherapeutic targetability of CNN3 in glioma patients using bioinformatics approach.

**Methods:**

CNN3 expression in glioma was analyzed based on GEO and TCGA datasets. Gene expression profiling with clinical information was employed to investigate the correlation between clinicopathological features of glioma patients and relative CNN3 expression levels. Survival analysis was conducted using Kaplan-Meier analysis and the Cox proportional-hazards regression model. Gene set enrichment analysis was conducted to select the pathways significantly enriched for CNN3 associations. Correlations between inflammatory activities, immune checkpoint molecules and CNN3 were probed by gene set variation analysis, correlograms, and correlation analysis.

**Results:**

CNN3 was enriched in gliomas, and high expression of CNN3 correlated with worse clinicopathological features and prognosis. Associations between CNN3 and several immune-related pathways were confirmed using a bioinformatics approach. Correlation analysis revealed that CNN3 was associated with inflammatory and immune activities, tumor microenvironment, and immune checkpoint molecules.

**Conclusion:**

Our results indicate that high CNN3 expression levels predict poor prognosis, and CNN3 may be a promising immunotherapy target.

## 1 Introduction

Gliomas were identified to be the most intrinsic type of primary intracranial tumors, accounting for 27% of all CNS tumors and 80% of malignant tumors.^[1,2]^ According to the WHO classified gliomas based on histology, it was categorized into three principal groups: astrocytoma, oligodendroglioma and oligoastrocytoma.^[3]^ With the advancement of genomics, several molecular markers related to specific tumor phenotypes have been identified, such as IDH, PI3K, TERT, PTEN, ATRX, EGFR, PDGFR, and H3 histone family member 3A.^[4–6]^ However, reliable markers are still lacking in the prognosis of glioma. Hence, it is urgent to identify a marker serving as a valuable prognosis predictor for glioma.

The traditional treatments of gliomas include radiation therapy, surgical resection and/or chemotherapy. Immunotherapy, with its recent successes in treating malignant solid and hematological cancers, has become one of the most popular research hotspots in glioma treatment.^[7]^ Glioma microenvironment and immune checkpoint inhibitors are of great importance in the research of immunotherapy. By far, the most important immune checkpoint receptors are cytotoxic T-lymphocyte-associated antigen 4 (CTLA-4), indoleamine 2,3-dioxygenase, and programmed cell death protein 1 (PD-1).^[8]^ However, the researches of the associations between some identified markers and the microenvironment or immune checkpoint inhibitors are still limited. Thus, confirmed associations between them might provide new ideas for the immunotherapy in glioma.

The calponin family of actin-binding proteins contains three isoforms.^[9]^ The protein encoded by CNN3 has been proven to be a member of the family, and it regulates cytoskeletal organization and actomyosin interactions through highly efficient binding to F-actin.^[10]^ Intriguingly, previous researches have demonstrated that CNN3 plays a unique role in some malignant tumors, such as osteosarcoma, cervical carcinoma tumors, colorectal tumors, gastric tumors and colon tumors.^[9,11–14]^ It was revealed that CNN3 served as a potential oncogene in some tumors and may promote the proliferation, invasion, metastasis, and drug resistance in some malignant tumors.^[9,11–14]^ However, the associations between CNN3 and its clinicopathological characteristics, immune infiltration in glioma remain unclear. Therefore, our research aims to investigate the prognosis value and immunotherapeutic targetability of CNN3 in glioma patients.

## 2 Methods

### 2.1 Public data collection and analysis

The microarray data were available at https://www.ncbi.nlm.nih.gov/geo under the accession number GSE50161. The GPL570 platform (Affymetrix Human Genome U133 Plus 2.0 Array) was utilized. The RNA-seq datasets and clinical information of glioma patients were downloaded from the TCGA databases (https://cancergenome.nih.gov/). Both low-grade glioma (LGG) and Glioblastoma datasets (HTSeq-FPKM) were encompassed. The relationships between CNN3 expression, clinicopathologic features and immune infiltration were further analyzed.

### 2.2 Bioinformatics approaches

GSEA was conducted to seek out differentially enriched biological pathways between samples with low and high CNN3 expression groups. Gene expression measurements of all tissues were transformed into scores for inflammatory response metagenes by GSVA. Correlograms then confirmed correlations between CNN3 and those metagenes.

### 2.3 Statistical analysis

A descriptive statistical analysis of the clinical and molecular features of glioma patients in the TCGA database was performed. The Shapiro–Wilk test was used to check the variables’ normality. Student's t-test was used to compare normal distributed variables, and Mann–Whitney U-test was used for non-normal distributed variables. Logistic regression analysis was applied to analyze the relationships between CNN3 and clinicopathological features of glioma patients. The prognostic significance of several variables including CNN3 expression related to overall survival in glioma patients was evaluated by the Kaplan-Meier and Cox regression methods. The correlation among CNN3 expression level, immune cell type, inflammatory type and immune checkpoint molecules was detected by classical correlation analysis. R software was utilized to conduct heatmap, circcos, and corrgram functions. All statistical analyses were performed using SPSS 25.0, GraphPad Prism 8.0 and R software 3.6 statistical software, and *P*-value < .05 was considered statistically significant.

## 3 Results

### 3.1 CNN3 expression was upregulated in glioma tissues

**Figure 1:**
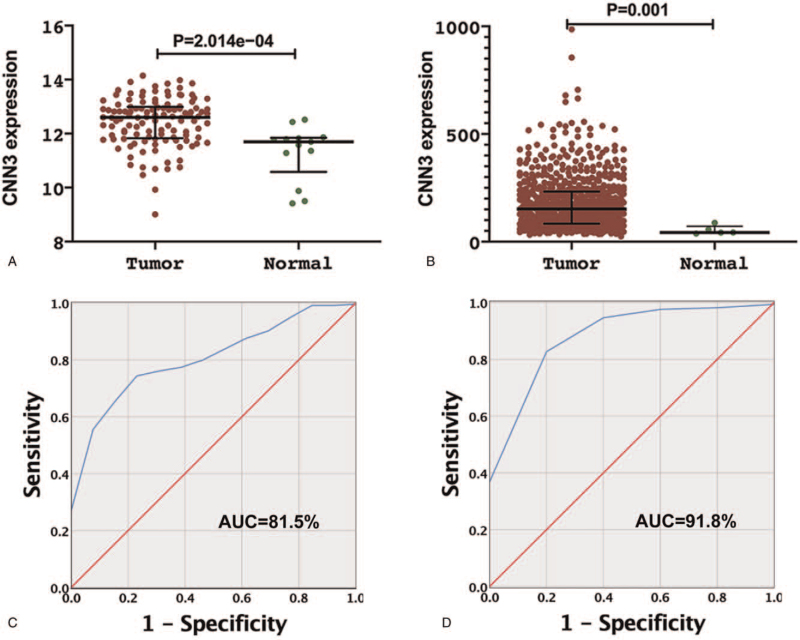
CNN3 expression levels in glioma and normal samples (A, GEO database; B, TCGA database). The predictive value of CNN3 for glioma according to the ROC curves (C, GEO database; D, TCGA database).

CNN3 expression in normal brain and glioma tissues was analyzed according to the RNA sequencing data. In the GEO cohort, the expression levels of CNN3 in glioma tissues were significantly higher than it in normal brain tissues (Fig. 1A, *P* < .001). The result was well confirmed in the GSE50161 dataset from the TCGA cohort (Fig. 1B, *P* < .001). To further verify this finding, ROC curves for CNN3 expression and two sample types are performed. The areas under the curves (AUC) are 81.5% in the GEO database and 91.8% in the TCGA database (Fig. C, D). These statistical results suggest that CNN3 might serve as a diagnostic biomarker for glioma.

### 3.2 Patient characteristics

The data of 1114 cases in the TCGA database were analyzed. Patient characteristics are summarized in Table 1. Of the 1114 cases, 651 (58.4%) were male, and 460 (41.3%) were female. The median patient age was 52 years (range 9–89 years). According to 2007 WHO classification, 249 patients had grade II glioma, 265 patients had grade III glioma, and 596 patients had grade IV glioma. The cohort consisted of 191 oligodendrogliomas, 196 astrocytomas, 596 Glioblastomas, and 130 oligoastrocytomas. Four cases were not provided with any histological information. Of the 1114 cases, 125 patients were examined for IDH mutation and identified 91 (8.17%). The number of patients with a Karnofsky performance score (KPS) of < 80 was 151 (13.6%), and the number of patients with a KPS score of ≥80 was 584 (52.4%). About tumor status, 783 (70.3%) cases had tumors, and 209 (18.8%) were tumor-free. At the latest follow-up, 539 (48.4%) patients died, and 570 (51.2%) were still alive.

**Table 1:** Patient characteristics.

Variable		Patient number	Centage (%)
Age (yr)	(9,89)	Median 52	
Gender	Men	651	58.4
	Women	460	41.3
	Unknow	3	0.27
WHO grade	G2	249	22.4
	G3	265	23.8
	G4	596	53.5
	Unknow	4	3.6
Histological type	Astrocytomas	196	17.4
	Oligodendrogliomas	191	17.2
	Oligoastrocytomas	130	11.7
	Glioblastoma	596	53.5
	Unknow	4	0.27
IDH mutation	YES	91	8.17
	NO	34	3.05
	Unknow	989	88.8
KPS	<80	151	13.6
	≥80	584	52.4
	Unknow	379	34.0
Tumor status	Tumor free	209	18.8
	With tumor	783	70.3
	Unknow	122	11.0
Vital status	Alive	570	51.2
	Dead	539	48.4
	Unknow	5	0.45

KPS = karnofsky performance score; IDH mutations = isocitrate dehydrogenase mutations.

### 3.3 Associations between CNN3 expression levels and clinicopathological features

**Figure 2:**
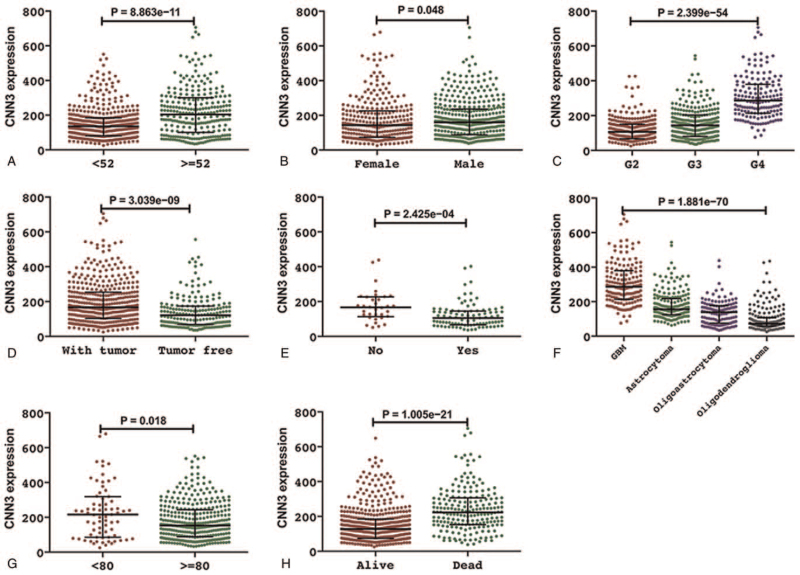
Associations between CNN3 expression and clinicopathological characteristics (TCGA database; A, age; B, gender; C, grade; D, tumor status; E, IDH mutation; F, histological type; G, KPS; H, vital status).

Statistical analysis was conducted to probe associations between CNN3 expression and clinicopathological features. As shown in Figure 2, high CNN3 expression level was related to older age (*P* < .001), male (*P* = .048), higher tumor grade (*P* < .001), lower KPS score (*P* = .018), and status of with tumor (*P* < .001). In addition, IDH mutation (*P* < .001), vital status (*P* < .001) and histological type (*P* < .001) were also significantly related to CNN3 expression.

Table 2 lists the results of logistic regression analyses. As it shows, high CNN3 expression is significantly relevant to age (*P* < .001), gender (*P* = .03), WHO grade (*P* < .001), histological type (*P* < .001), IDH mutation (*P* = .002), KPS (*P* = .01), tumor status (*P* < .001) and vital status (*P* < .001). Herein, we can conclude that CNN3 may be an oncogene for glioma, and high CNN3 expression may contribute to a relatively poor prognosis.

**Table 2:** Relationships between CNN3 expression level and clinicopathological features using logistic regression.

Clinical characteristics	OR in CNN3 expression	95% CI	*P*-value
Age			
≥52 vs <52	2.83	2.05–3.92	<.001
Gender			
Male vs Female	1.41	1.04–1.92	.03
WHO grade			
III vs II	2.81	1.93–4.13	<.001
IV vs II	70.02	33.24–172.49	<.001
Histology type			
Glioblastoma vs Astrocytoma	19.29	9.18–47.36	<.001
Glioblastoma vs Oligodendroglioma	121.34	55.02–310.21	<.001
Glioblastoma vs Oligoastrocytomas	33.00	15.23–82.90	<.001
IDH mutation			
No vs Yes	4.05	1.75–10.11	.002
KPS			
<80 vs ≥80	1.94	1.15–3.31	.01
Tumor status			
With tumor vs Tumor free	2.62	1.84–3.77	<.001
Survival condition			
Dead vs Alive	4.82	3.34–7.04	<.001

CI = confidence interval, KPS = karnofsky performance score, OR = odds ratio.

### 3.4 High expression of CNN3 predicted unfavorable prognosis

**Figure 3:**
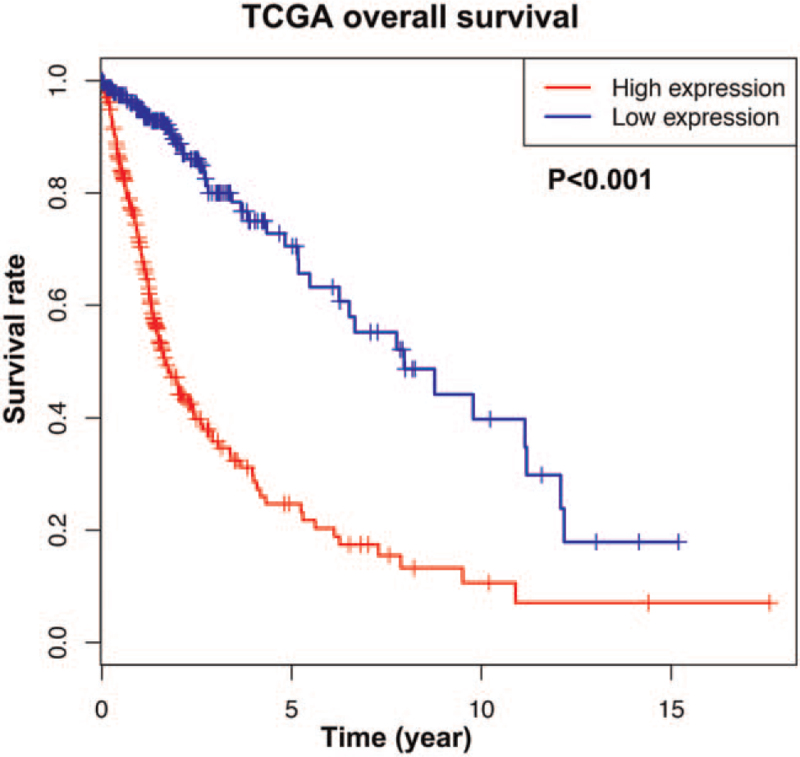
Overall survival for glioma patients between high and low CNN3 expression cohorts (TCGA database).

The predictive value of CNN3 in glioma patients was evaluated by the Kaplan-Meier curve plotted based on the TCGA database. It showed that high CNN3 expression predicted worse prognosis (Fig. 3, *P* < .001).

The prognosis value of CNN3 in glioma was further investigated by univariate and multivariate analyses (Table 3). A total of 371 cases with sufficient variables were selected and analyzed. The univariate Cox proportional hazards regression suggested that high CNN3 expression was related to worse overall survival (HR = 1.00, 95% Cl 1.00–1.01, *P* < .001). Besides, age (HR = 1.07, 95% Cl 1.05–1.08, *P* < .001), grade (HR = 4.66, 95% Cl 3.48–6.25, *P* < .001), KPS (HR = 0.95, 95% Cl 0.94–0.96, *P* < .001), histological type (HR = 2.45, 95% Cl 2.00–3.00, *P* < .001), tumor status (HR = 37.06, 95% Cl 5.18–265.21, *P* < .001) also correlated with overall survival. In the Multivariate Cox regression analysis, histological type (HR = 2.17, 95% Cl 1.71–2.74, *P* < .001) and CNN3 (HR = 1.00, 95% Cl 1.000–1.003, *P* = .046) expression in glioma were both independently associated with overall survival. Therefore, CNN3 might be identified as a prognostic molecular marker for glioma according to the results above.

**Table 3:** Univariate (A) and multivariate (B) cox regression of overall survival in TCGA.

Variable	HR (95% CI)	*P*-value
**A.**		
Age	1.07 (1.05–1.08)	<.001
Gender	0.98 (0.70–1.38)	.92
KPS	0.95 (0.94–0.96)	<.001
Grade	4.66 (3.48–6.25)	<.001
Tumor status	37.06 (5.18–265.21)	<.001
Vital status	846187177.6 (0-Inf)	.99
Histological type	2.45 (2.00–3.00)	<.001
CNN3	1.00 (1.00–1.01)	<.001
**B.**		
Histological type	2.17 (1.71–2.74)	<.001
CNN3	1.00 (1.000–1.003)	.046

HR = hazard rate, KPS = karnofsky performance score.

### 3.5 CNN3-related signaling pathways

**Figure 4:**
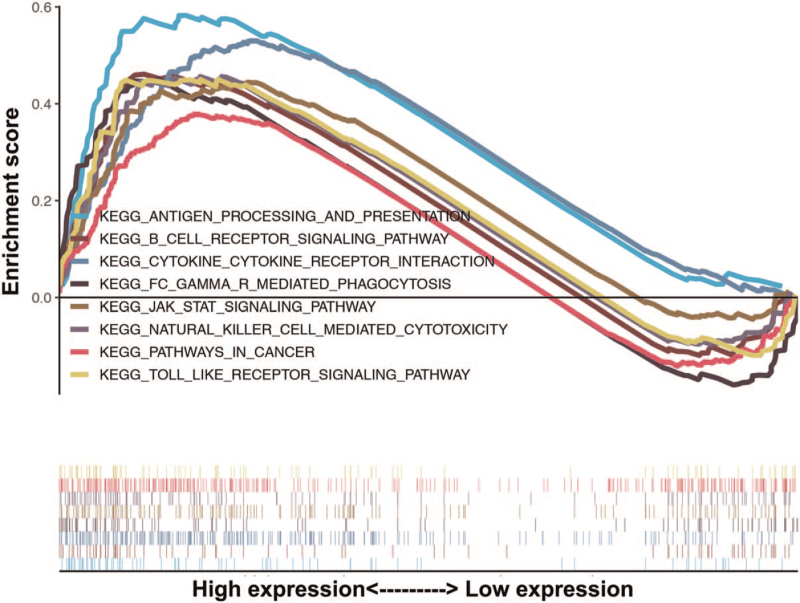
GSEA for CNN3.

GSEA was utilized to explore the links between CNN3 expression and signaling pathways. Statistically significant values are expressed as normalized P < 0.05, and the results are shown in Figure 4 and Table 4. It was observed that the B cell receptor signaling pathway, antigen processing and presentation, Fc gamma R-mediated phagocytosis, cytokine-cytokine receptor interactions, natural killer cell-mediated cytotoxicity, the JAK-STAT signaling pathway and the Toll-like receptor signaling pathway were significantly enriched in patients with a higher CNN3 expression. The signaling pathways mentioned above are mostly involved in inflammatory and immune responses.

**Table 4:** Gene sets enriched in the high expression phenotype group.

Gene set name	NES	NOM *P*-value
KEGG_B_CELL_RECEPTOR_SIGNALING_PATHWAY	1.65	.04
KEGG_ANTIGEN_PROCESSING_AND_PRESENTATION	1.77	.02
KEGG_CYTOKINE_CYTOKINE_RECEPTOR_INTERACTION	1.80	.01
KEGG_FC_GAMMA_R_MEDIATED_PHAGOCYTOSIS	1.67	.03
KEGG_JAK_STAT_SIGNALING_PATHWAY	1.66	.03
KEGG_NATURAL_KILLER_CELL_MEDIATED_CYTOTOXICITY	1.67	.02
KEGG_PATHWAYS_IN_CANCER	1.56	.03
KEGG_TOLL_LIKE_RECEPTOR_SIGNALING_PATHWAY	1.59	.04

FDR q-val = false discovery rate q-value, NES = normalized enrichment score, NOM p-val = normalized *P*- value.

### 3.6 CNN3-related inflammatory activities

**Figure 5:**
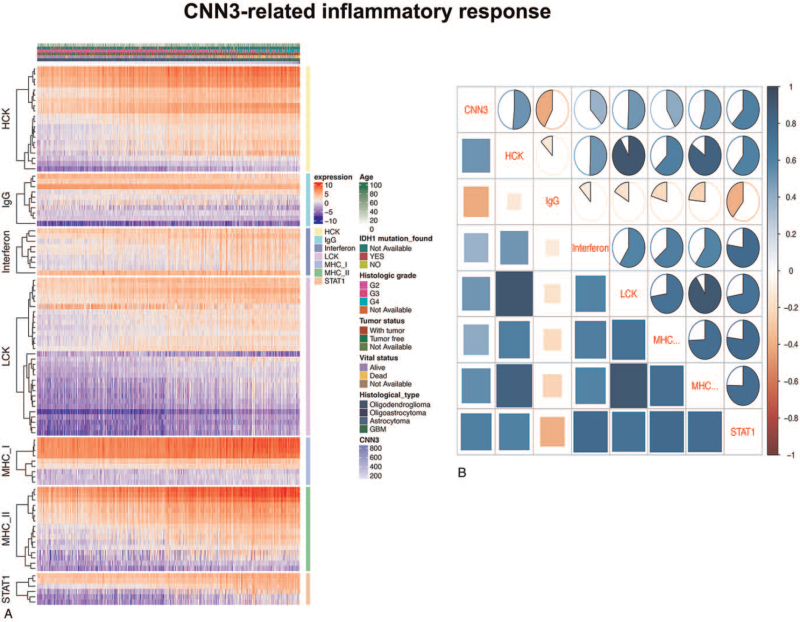
Inflammatory activities related to CNN3. A, Heat map related to CNN3 expression, clinicopathological characteristics and several metagenes based on the TCGA database. B, Correlograms between the GSVA enrichment scores of these metagenes and CNN3 expression. Blue stands for a positive correlation and red denotes a negative correlation.

To better understand CNN3-related inflammation activities, we analyzed seven clusters including 104 genes, which representing different immune and inflammatory activities for research. The expression of CNN3 has a positive correlation with LCK, MHC I, HCK, interferon, MHC II, and STAT1 clusters. However, among the seven clusters, the IgG cluster was observed with a negative correlation. Gene expression data was then converted into enrichment scores using GSVA to confirm the results above. According to the correlograms (Fig. 5B), the associations between CNN3 and seven metagenes were in accordance with the results above.

### 3.7 Correlations between CNN3 and infiltrating immune cells

**Figure 6:**
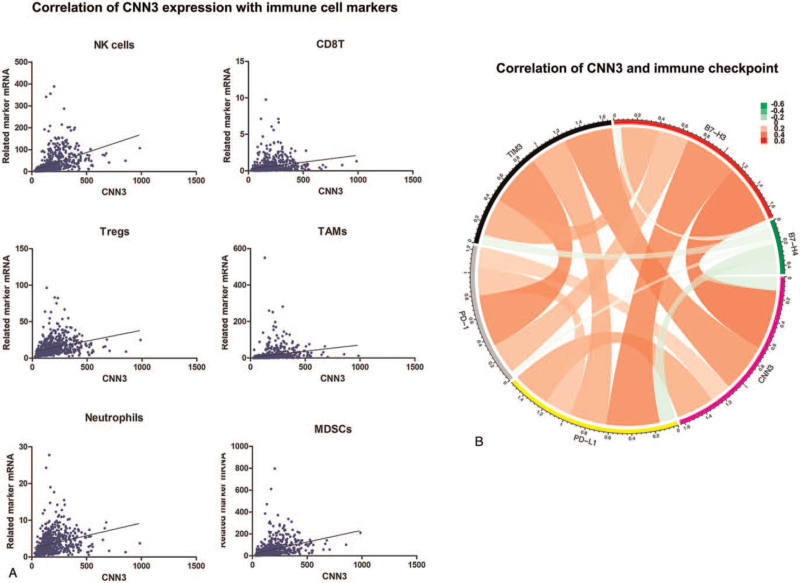
A, Associations between immune cell markers and CNN3 expression. B, Correlation between CNN3 and immune checkpoints using TCGA databases.

The associations between CNN3 and six immune cell types including tumor-associated macrophages (TAMs), CD8+ T cells, regulatory T cells (Tregs), natural killer (NK) cells, myeloid-derived suppressor cells (MDSCs) and neutrophils were investigated. These immune cells were often recruited into the tumor microenvironment. As shown in Figure 6A, CNN3 expression had a positive correlation with six immune cell-specific markers. It revealed that glioma tumors with high CNN3 expression might recruit more immune cells into the tumor microenvironment than gliomas with low CNN3 expression.

### 3.8 Associations between CNN3 and immune checkpoint molecules

Pearson correlation analysis was utilized to analyze the associations between CNN3 and immune checkpoint molecules—PD-1, PD-L1, B7-H4, B7-H3, and TIM-3 in TCGA data. As shown in Figure 6B, B7-H3, TIM3, PD-1 and PD-L1 have a significantly positive relationship with CNN3, which indicates that CNN3 might be an immunotherapeutic target in glioma.

## 4 Discussion

In the current study, CNN3 was observed to have higher expression levels in glioma samples than normal tissues in the TCGA and GEO datasets. The RNA sequencing of 1114 glioma patients from the TCGA database was retrospectively analyzed. It was indicated that the expression levels of CNN3 were related to the clinicopathological features including age, gender, histological type, WHO grade, IDH mutation, KPS, tumor status and vital status. In addition, the Kaplan-Meier analysis illustrated that high CNN3 expression levels predicted worse overall survival in glioma patients. Moreover, the univariate and multivariate Cox regression analyses further revealed that CNN3 was an independent prognostic biomarker for glioma.

Gene GSEA analyses suggested that CNN3 was significantly enriched in inflammatory and immune-related pathways and biological processes. To further investigate the inflammatory activities related to CNN3, heatmaps comprising seven clusters of immune-related genes were utilized. It indicated that CNN3 had a positive correlation with the interferon, HCK, LCK, MHC II, MHC I and STAT1 metagenes, but a negative correlation with IgG. A correlogram using GSVA analysis was further generated to verify the results. Furthermore, the canonical correlation analysis revealed that CNN3 has a positive correlation with six essential immune cells (Tregs, TAMs, MDSCs, CD8+T cells, NK cells, and neutrophils). Besides, associations between CNN3 and several remarkable immune checkpoint molecules containing B7-H3, B7-H4, PD-L1, PD-1 and TIM3 were confirmed through circus plots. The results above proposed that CNN3 was putatively related to the immune microenvironment of gliomas.

Previous studies have shown that CNN3 played essential roles in various types of tumors. Dai et al reported that high expression of CNN3 was observed in osteosarcoma tissues and cell lines, and silence of CNN3 expression can inhibit subcutaneous tumor growth and lung metastasis in vivo.^[11]^ Besides, CNN3 mRNA expression was also found to be elevated in cervical cancer tissues, and knockdown down of it was indicated to display decreased growth and metastasis in xenografted tumo.^[14]^ Nair et al demonstrated that CNN3 was related to lymph node metastasis and antagonistic effects in colon cancer.^[9]^ Moreover, CNN3 was also found to promote cell invasion and agonistic effects to doxorubicin in gastric cancer.^[12]^ In the current study, it was confirmed that high CNN3 expression levels in glioma associated with worse clinicopathologic features and prognosis. The above findings implied that CNN3 might be a therapeutic target for tumors.

The immune microenvironment of tumors is a new concept related to the mutual resistance of immunity and malignant tumor cells.^[15]^ Pro-inflammatory cytokines produced in the tumor microenvironment was reported to inhibit the anti-tumor immunity and promote the progression of tumor.^[16]^ In glioma, tumor cells can facilitate its proliferation, invasion and drug-resistance through adjusting the microenvironment.^[17]^ It is demonstrated that some cytokines, which can introduce the infiltration of immune cells (CD8+T cells, Tregs, NK cells, TAMs, MDSCs, and neutrophils, etc.), could be produced by glioma cells.^[17]^ In the current study, we were surprised to find that CNN3 had a positive correlation with these immune cells. Therefore, it is proposed that CNN3 may promote gliomas’ progression through its impact on these immunosuppressive cells. However, more detailed and precise research is needed to validate this prediction and uncover the underlying mechanisms of the mutual interactions between them.

Recently, immunotherapy with immune checkpoint inhibitors is advancing rapidly and has attracted attention in cancer treatment.^[18,19]^ So far, immunotherapy has shown great efficacy in various types of tumors, and researchers are paying increased *attention* to immunotherapy for glioma patients.^[20]^ However, glioma and glioblastoma have remained refractory to monotherapy with ICIs.^[8,21]^ Meanwhile, it was suggested that combination immunotherapies offer more potent anti-tumor activity.^[22]^ Previous research indicated that dual blockade of PD-1-LAG-3 or PD-1-CTLA-4 pathways had greater efficacy to control murine ovarian tumor growth than blocking PD-1 alone and that triple blockade of PD-1-CTLA-4-LAG-3 pathways was superior if PD-1 pathway was completely blocked.^[23]^ Khair et al also confirmed that dual blockade of checkpoint molecules PD-1/PD-L1 and CTLA-4 had shown great success in prompting immune responses against malignant tumors and may lead to tumor regression in many melanoma patients.^[24]^ These findings generated increasing interest in combination immunotherapies in glioma and glioblastoma patients. In this study, we surprisingly found out that CNN3 had a significant correlation with several immune checkpoint molecules containing PD-1, TIM-3, PD-L1 and B7-H3 (Fig. 6). It provides a new idea to further investigate the combination immunotherapy involving CNN3 and these prominent immune checkpoint molecules.

The limitations of this study are listed as follows: Firstly, patients included in the univariate and multivariate Cox regressions were limited because some patient data may not be available. Secondly, the clinical sample size in the matched control group was relatively small. Thus, further research involving more negative samples is needed. Finally, further experimental investigations are required to uncover the mechanism of CNN3 overexpression in glioma tissues and explore the relationships among CNN3, immune microenvironment, and glioma progression.

## 5 Conclusion

Overall, our study indicated that CNN3 was overexpressed in glioma tissues. High expression levels of CNN3 correlates with a higher degree of malignancy of glioma samples and poorer prognosis. It was also demonstrated that CNN3 was associated with the inflammatory and immune activities of glioma and was involved in checkpoint molecules. In summary, our results indicate that a high CNN3 expression level predicts poor prognosis, and CNN3 may be a promising immunotherapy target.

## Acknowledgments

I would like to express my gratitude to all those who helped me during the writing of this thesis.

## Author contributions

**Conceptualization:** Hao Xu.

**Data curation:** Peng Lv, Hao Xu, Song-shan Chai.

**Formal analysis:** Hao Xu.

**Investigation:** Hao Xu, Jia-jing Wang.

**Methodology:** Hao Xu, Hao Xu, Jia-jing Wang.

**Project administration:** Song-shan Chai, Jia-jing Wang.

**Resources:** Songshan Chai, Peng Lv, Song-shan Chai.

**Software:** Hao Xu.

**Supervision:** Songshan Chai.

**Validation:** Jia-jing Wang.

**Visualization:** Peng Lv, Jia-jing Wang.

**Writing – original draft:** Hao Xu, Jia-jing Wang.

**Writing – review & editing:** Hao Xu, Jia-jing Wang.
